# Supplementary material for: The causal relationship between the human gut microbiota and pyogenic arthritis: a Mendelian randomization study
Source: Front Cell Infect Microbiol. 2024 Nov 26;14:1452480. doi: 10.3389/fcimb.2024.1452480 (PMC11629706; doi:10.3389/fcimb.2024.1452480)
Supplement: Supplementary file 1 [file DataSheet1.zip › mendelian test/ebi-a-GCST90027521.csv_leave_one_out.pdf]

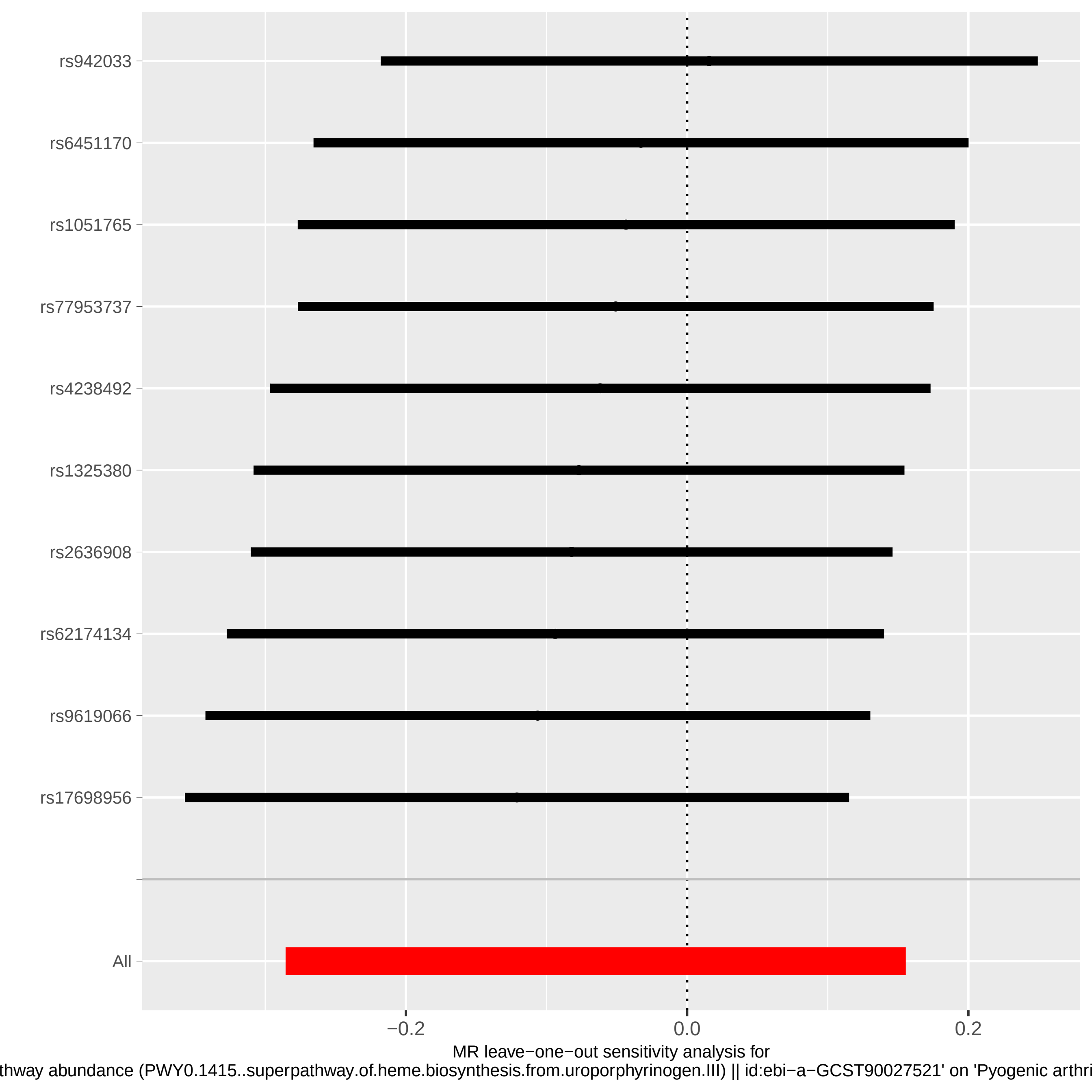
rs942033
rs6451170
rs1051765
rs77953737
rs4238492
rs1325380
rs2636908
rs62174134
rs9619066
rs17698956
All
−0.2
0.0
0.2
MR leave−one−out sensitivity analysis for
thway abundance (PWY0.1415..superpathway.of.heme.biosynthesis.from.uroporphyrinogen.III) || id:ebi−a−GCST90027521' on 'Pyogenic arthri
